# Successful Outcome of a Triplet Gestation in a Patient with a History of an Open Maternal-Fetal Surgery

**DOI:** 10.1155/2012/131467

**Published:** 2012-12-18

**Authors:** Kathleen A. Rivers, Paul W. Whitecar, Cristóbal S. Berry-Cabán

**Affiliations:** ^1^Department of Maternal Fetal Medicine, Womack Army Medical Center, Fort Bragg, NC 28310, USA; ^2^Clinical Investigation Services, Womack Army Medical Center, Fort Bragg, NC 28310, USA

## Abstract

Reproductive outcomes in women after pregnancy complicated by an open maternal-fetal surgery (OMFS) are limited. A review of the medical literature reveals only isolated cases of successful multiple pregnancies, and there are no prior documented cases of successful triplet gestations following OMFS. We report the delivery of a triplet gestation at 34-week gestation in a patient with a history of previous OMFS.

## 1. Introduction

Reproductive outcome data on women who have previously undergone an open maternal-fetal surgery (OMFS) is limited. Wilson et al. reported on the reproductive outcomes of a cohort of 93 women. Among this group, there were 47 pregnancies beyond 20-week gestation; the rate of uterine rupture and dehiscence was 14%. Wilsons' study included two twin pairs [1]. A Medline search (1966–2011) using the search terms “multiple gestations” and “open maternal-fetal surgery” revealed no cases of successful triplet or higher order pregnancies in women with prior OMFS.

## 2. Case

A 37-year-old patient G5P1122 conceived triplets following assisted reproductive techniques. She had previously undergone multiple uterine surgeries including a low transverse cesarean delivery in her second pregnancy. She underwent OMFS for the repair of a fetal meningomyelocele in her third pregnancy that resulted in a preterm delivery by classical cesarean at 26 weeks due to preterm labor. During pregnancy number 3, she had participated in the Management of Myelomeningocele Study (MOMS) and underwent OMFS at 24 weeks at The Children's Hospital of Philadelphia. In pregnancy no. 4, the patient conceived a triamnionic, trichorionic triplet gestation after assisted reproductive techniques. Her pregnancy course was complicated by cervical shortening and suspected incompetent cervix for which she underwent a McDonald cerclage at 20-week gestation. Due, in part, to concerns for uterine rupture, at 32-week gestation, she was admitted for bed rest and observation. She completed a course of betamethasone for fetal lung maturity. She underwent a repeate cesarean delivery at 34-week gestation for triplet gestation that was productive of three healthy infants. Dense adhesions were noted and precluded a planned bilateral tubal ligation. The uterine scar appeared intact at the time of surgery.

## 3. Discussion

Despite intervention, approximately from 14% to 35% of children born with myelomeningocele fail to survive past 5 years of age. The higher mortality rate is dependent on brainstem dysfunction secondary to hindbrain herniation (Arnold-Chiari malformation). Only about half of patients are able to function independently as adults with an intelligence quotient less than 80 in about 70% of patients. Many are confined to wheel chairs, troubled by bowel and bladder control problems, and have hydrocephalus requiring shunts and needing multiple revisions [2, 3].

Prior to 1997, treatment of myelomeningocele was limited to corrective surgery in the postnatal period for the infant, followed by lifelong supportive care. Without protective tissue coverage, secondary destruction of the exposed spinal cord may occur throughout gestation and delivery. A proposed “two hit hypothesis” states that the neural damage associated with myelomeningocele may be a result of a relatively normal spinal cord that becomes secondarily damaged by a combination of amniotic fluid exposure, trauma, and hydrodynamic pressure. It is anticipated that secondary damage could be eliminated through the use of OMFS [4]. 

The management of myelomeningocele study (MOMS) was terminated in December 2010 based on the efficacy of prenatal surgery in the 183 pregnancies studied. Prenatal surgery was found to reduce the need for postnatal shunting and resulted in improved motor outcomes at 30 months for the affected infants [5, 6]. 

In spite of the benefits, prenatal surgery is not without increased risk of morbidity for the infants and the mother. Wilson et al. made the recommendation that women with a history of OMFS delay subsequent pregnancies for a minimum of 18 to 24 months from the date of delivery of the index pregnancy. They also recommended to repeat cesarean section at 36 weeks without documentation of fetal lung maturity in the subsequent pregnancies [1]. 

Women undergoing elective cesarean section have been reported to have a 3-fold higher risk of mortality compared with women who deliver vaginally_._ The reported obstetric risk in subsequent pregnancy after OMFS is a major prenatal counseling concern with the quoted risk of uterine dehiscence and rupture of 14%, respectively. Despite the report that the concern for uterine rupture caused anxiety and depression in some cases, more than 95% of mothers said they would choose an open maternal-fetal surgery again if it was an option.

 Fetal anesthesia risks were reported in at least one study. Children with 2 exposures to anesthesia before the age of 4 were 59% more likely than unexposed children to be diagnosed with learning disabilities [1]. 

In summary, our case reports a successful outcome in what is believed to be the first documented triplet gestation in a patient with prior OMFS. Despite the favorable outcome, this case highlights the need for further research in reproductive outcomes in this subset of women. With advances in OMFS for lethal and nonlethal anomalies, followup of reproductive outcomes is a continued necessity to determine the fetal and maternal risk benefit for ethical and medical sound patient counseling.
